# GaN Nanowire Array for Charge Transfer in Hybrid GaN/P3HT:PC_71_BM Photovoltaic Heterostructure Fabricated on Silicon

**DOI:** 10.3390/ma13214755

**Published:** 2020-10-24

**Authors:** Giorgi Tchutchulashvili, Sergij Chusnutdinow, Wojciech Mech, Krzysztof P. Korona, Anna Reszka, Marta Sobanska, Zbigniew R. Zytkiewicz, Wojciech Sadowski

**Affiliations:** 1 Faculty of Applied Physics and Mathematics, Gdansk University of Technology, Gabriela Narutowicza 11/12, 80-233 Gdansk, Poland; wsadowski@pg.edu.pl; 2 Institute of Physics, Polish Academy of Sciences, al. Lotników 32/46, 02-668 Warsaw, Poland; chusnut@ifpan.edu.pl (S.C.); reszka@ifpan.edu.pl (A.R.); sobanska@ifpan.edu.pl (M.S.); zytkie@ifpan.edu.pl (Z.R.Z.); 3 Faculty of Physics, University of Warsaw, Pasteura 5, 02-093 Warsaw, Poland; Wojciech.Mech@fuw.edu.pl (W.M.); kkorona@fuw.edu.pl (K.P.K.)

**Keywords:** P3HT, PCBM, GaN nanowires, hybrid photovoltaics, silicon, organic–inorganic, bulk heterojunction

## Abstract

We demonstrate that a GaN nanowire array can be used for efficient charge transfer between the organic photovoltaic layer and silicon in a Si/GaN/P3HT:PC_71_BM inverted hybrid heterostructure. The band alignment of such a material combination is favorable to facilitate exciton dissociation, carrier separation and electron transport into Si. The ordered nature of the GaN array helps to mitigate the intrinsic performance limitations of the organic active layer. The dependence of photovoltaic performance enhancement on the morphology of the nanostructure with nanowire diameters 30, 50, 60, 100 and 150 nm was studied in detail. The short circuit current was enhanced by a factor of 4.25, while an open circuit voltage increase by 0.32 volts was achieved compared to similar planar layers.

## 1. Introduction

**Figure 1:**
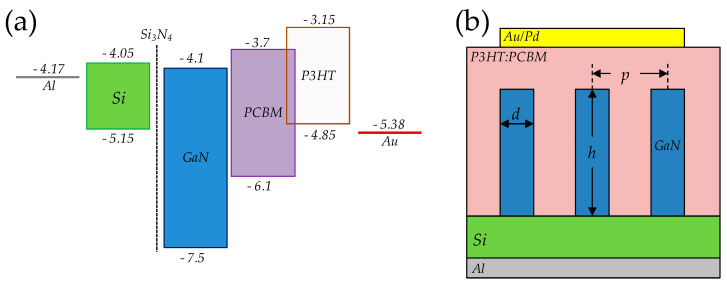
(**a**) Theoretical energy band alignment of Si/GaN NW/P3HT:PC_71_BM hybrid heterostructure. Energy levels are in eV. (**b**) Structure schematic with designated nanostructure diameter *d*, height *h* and pitch *p*. Relative distances not to scale.

Hybrid organic–inorganic solar cells based on inorganic nanowires (NWs) embedded in photosensitive polymer films have been the focus of research on ways to enhance the power conversion efficiency of bulk heterojunction (BHJ) organic photovoltaic cells by the simultaneous application of several strategies: enhanced light absorption by NW arrays, improved charge separation, carrier collection and transport in inorganic acceptors [1,2]. One of the most researched light-sensitive polymers, the poly(3-hexylthiophene-2,5-diyl) in mixture with [6,6]-Phenyl C_71_ butyric acid methyl ester acceptor, commonly referred to as P3HT:PC_71_BM, has been a workhorse for organic photovoltaics research due to its light absorption properties and optimum band gap of 1.9 eV [3]. Various inorganic NWs have been implemented as organic acceptor substitutes in BHJ solar cells [4,5,6], leading to power conversion efficiency optimization. Though the GaN synthesis is well understood, only a few attempts have been made to create GaN/P3HT hybrid structures. The application of a nanostructured GaN acceptor will take advantage of the unique properties of GaN. For example, Calarco et al. report decreased surface recombination in GaN nanowires due to band bending near the surface [7]. The theoretical alignment of energy levels [8,9] presented in Figure 1a shows that the Si/GaN/P3HT:PC_71_BM system forms a cascade type II heterojunction, which permits drift of the carriers generated through light absorption in P3HT. The energetic difference between the lowest unoccupied molecular orbital (LUMO) of P3HT and the conductive band edge of GaN exceeds the binding energy of excitons (E_b_ = 0.3–0.7 eV) in P3HT [10,11], which allows for efficient charge separation. The objective of this research is to exploit the properties of GaN for light harvesting and carrier transport. A nanowire array could be used as a light guide to increase illumination for lower layers of the polymer. Together with the light scattering effect, this leads to increased quantum efficiency. Additionally, lower energy photons, not absorbed by P3HT, could be harvested in the silicon substrate, leading to increased power-converting efficiency in the multilayer structure after proper optimization and current matching have been conducted. The other objective is to achieve a large junction area and a current path through the GaN nanowire for efficient electron collection.

Several research teams have reported on the fabrication and characterization of GaN/P3HT structures. The electrical properties of the GaN/P3HT interface have been studied [8,12]. Studies of planar GaN/P3HT layers [13], GaN nanoparticle/P3HT [14], porous GaN/P3HT [15] and GaN quantum dot/P3HT [16] hybrid layers utilizing different synthesis techniques have also been reported. Photovoltaic performance parameters J_sc_ = 3.5 mA/cm at V_oc_ = 0.71 V have been achieved for GaN nanoparticle/P3HT composite films [14]. Recently, we reported the fabrication and characterization of a Si/GaN NW/P3HT hybrid together with detailed analysis of the charge transfer mechanism in such a structure [17].

In this study, a hybrid Si/GaN NW/P3HT:PC_71_BM structure was fabricated on photovoltaic-grade silicon (Figure 1b). The systematic investigation of photovoltaic performance parameters’ dependence on the morphology of the ordered GaN nanowire array is reported, together with Mott–Schottky analysis results. These data could be useful for hybrid solar cell design, especially in the context of creating the top cell in a Si-based multilayer structure or using bulk silicon to recover non-absorption losses in the top layer.

## 2. Materials and Methods 

### 2.1. Growth of GaN Nanowires

A series of GaN NW growths was performed on monocrystalline 3″ Si (111) from Si-Mat Silicon Materials by plasma-assisted molecular beam epitaxy (PAMBE) technique using Riber Compact21 reactor equipped with the standard Knudsen gallium cell. Active nitrogen species were produced by radio frequency Addon nitrogen plasma source controlled by an optical light sensor [18]. After thermal desorption of native silicon oxide, 2 nm silicon nitride layer was formed by exposing the substrate to nitrogen plasma flux for 15 min at 750 °C [19]. Then, catalyst-free NW growth was conducted under N-rich conditions with III/V flux ratio equal to 0.5. Structures with average nanowire diameters 30, 50, 60, 100 and 150 nm (designated as D30 to D150) were synthesized at growth temperatures in the range of 800–840 °C. Since at higher temperatures, Ga desorption from the surface increases, for fixed impinging Ga flux, the amount of gallium available for NW growth decreases. As shown by Fernández-Garrido et al. [20], this leads to slower lateral growth and, consequently, to thinner NWs. For all samples, the growth time was adjusted to fabricate 200 nm long NWs. More details on the growth procedure have been published elsewhere [21,22]. Silicon was used as an n-type dopant for GaN. The estimated doping level was 5 × 10^18^ cm^−3^ based on the silicon incorporation into reference planar GaN samples. Results of energy-dispersive X-ray spectroscopy and X-ray diffraction characterization of so-produced GaN NWs have been reported elsewhere [17,23,24]. Finally, 500 nm thick planar GaN layer with the same doping level was grown directly on Si(111) [25]. It was then used for fabrication of reference planar hybrid devices.

### 2.2. Preparation of the Organic Blends

Regioregular P3HT with 54,000–75,000 g/mol average molecular weight was mixed with PC_71_BM in chloroform solution with concentrations 15 and 10 mg/mL, respectively. After mixing for 24 h at 40°C, the blend was cooled to room temperature and filtered through the 0.45 μm syringe filter. All organic materials were purchased from Sigma-Aldrich (Darmstadt, Germany).

### 2.3. Structure Fabrication

All devices were prepared in a dry argon atmosphere glovebox by spin-coating a photoactive layer of P3HT:PC_71_BM (3:2 weight ratio in chlorobenzene) onto GaN NW arrays. Al bottom electrical contact to the back of the Si substrate was deposited previously. Then, films were annealed on a hotplate for 15 min at 150 °C. Upper Au/Pd contacts were prepared by radio-frequency sputtering in Ar. Transparency of the top contact was found to be equal to 20%. All investigated structures had active areas of 4 mm^2^. At least four devices were formed for each nanowire diameter and measurement results were averaged. More details of fabrication steps have been published previously [17].

## 3. Characterization

### 3.1. Scanning Electron Microscopy and Morphological Feature Analysis

Hitachi SU-70 field-emission scanning electron microscope (FE-SEM, Krefeld, Germany) operating at 5 keV was employed to study the morphology of GaN nanostructures. Raw SEM images taken with the definition of 0.5 nm/pixel were converted to binary maps and analyzed with the use of the open-source ImageJ software [26]. Statistical data about average NW diameter d, height h, pitch p, area fraction and aggregate particle perimeter data were collected using the particle analysis tool. Nanowires and their aggregates were approximated as ellipses and minor axes were taken as NW diameter. The free volume parameter used in the analysis was defined as a fraction of active layer volume unoccupied by NWs and was calculated by the following equation:

f = V_P3HT_/V_total_ = h(S_total_ − S_NW_)/hS_total_ = 1 − S_NW_/S_total_ (1)

where S_total_ is the total area of the SEM image being analyzed, S_NW_ is the aggregate area of nanowire bases measured in ImageJ and h is the average nanowire height. The specific surface parameter characterizes the additional junction area created by NWs. It is a factor by which the heterojunction area increases compared to the planar layer per unit area of the substrate. It was calculated using the following equation:

S = (S_total_ + h·P_NW_)/S_total_ = 1 + (h·P_NW_)/S_total_ (2)

where P_NW_ is aggregate nanowire perimeter measured in ImageJ.

### 3.2. Volt-Ampere Characteristics Measurement

Current density–voltage (J-V) characteristics were recorded on a PEQUEST solar simulator (Milford, CT, USA) outfitted with Tracer 2 software package. LOT Oriel 300-watt Xe lamp (Darmstadt, Germany) was used for AM1.5 solar spectrum simulation. Photovoltaic performance parameters were obtained from J-V dependences. Series and shunt resistance values were obtained by manual fitting of J-V curves.

### 3.3. Mott–Schottky Analysis

The capacitance–voltage curves were measured in a 4-terminal configuration using the Zurich Instruments MLFI 5 MHz lock-in amplifier with an impedance analyzer option (MF-IA) in the 2 V and 10 mA voltage and current range, respectively. The equivalent circuit was representative of parallel resistance and capacitance (R_p_ || C_p_). The heterostructure was investigated at a frequency of 2 kHz and voltage range of +/− 1 V at room temperature. Results were interpreted using standard Mott–Schottky analysis [27]. Built-in voltage (V_bi_) values were extracted from the intercepts of the linear part of 1/C^2^(V) plot with voltage axis V_bi_ = V_y=0_, and effective doping |N_a_−N_d_| was estimated from the slope of 1/C^2^ curve using the following relation:

$$|N_{a}-N_{d}|=\frac{2}{A^{2}e\varepsilon \varepsilon_{0}\cdot \Delta (1/C^{2})/\Delta V)}$$

where A is device area, e—elementary charge, ε—relative permittivity (equal 3 for P3HT:PCBM blend), ε_0_—dielectric permittivity of vacuum [28].

## 4. Results and Discussion

### 4.1. Nanostructure Morphology

**Figure 2:**
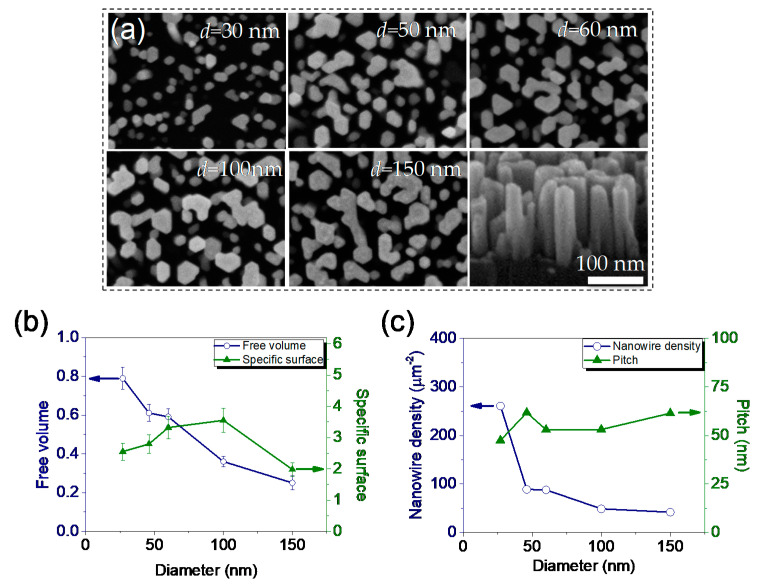
(**a**) Plan and bird’s eye view SEM images of GaN nanostructures with average diameters 30, 50, 60, 100 and 150 nm. Nanowire coalescence could be observed for d = 50−100 nm with a transition into nano-walls at d = 150 nm. Morphological features of GaN NWs: (**b**) Free volume (left axis, blue circles) and specific surface (right axis, green triangles) vs. average diameter; (**c**) Nanowire density per μm^2^ (left axis, blue circles) and pitch vs. average NW diameter (right axis, green triangles). Color online.

Plan and bird’s eye view SEM images of GaN nanostructures with average diameters d = 30, 50, 100 and 150 nm are shown in Figure 2a. The last panel in Figure 2a shows that GaN nanowires form ordered vertical arrays with a height of around h = 200 nm. Figure 2b shows the free volume of nanowire film (left axis, blue circles) and specific surface (right axis, green triangles) as a function of average diameter d. With the increase in d, the free volume of the nanostructure film decreases monotonously as more GaN material is added to the surface. On the contrary, the specific surface is distributed with a maximum of around 3 for d = 100 nm nanowires. After this, the value of the specific surface reduces due to coalescence, as for every two hexagonal nanowires which agglomerate along the single sidewall, the surface of the resulting agglomerate is reduced by 1/6th. Such a decrease in nanostructure specific surface could be observed after d = 100.

NW distribution density (left axis, blue circles) and pitch (right axis, green triangles) are shown in Figure 2c. NW density falls sharply between d = 30 and d = 50 nm and continues with a slight decrease after d = 60 nm. At the same time, pitch between nanostructures expands. A range of stable morphological parameters between d = 50 and d = 100 could be identified. The further increase in NW diameter above 100 nm causes free volume and total surface area to reduce due to NW coalescence.

### 4.2. Current–Voltage Characterization

**Figure 3:**
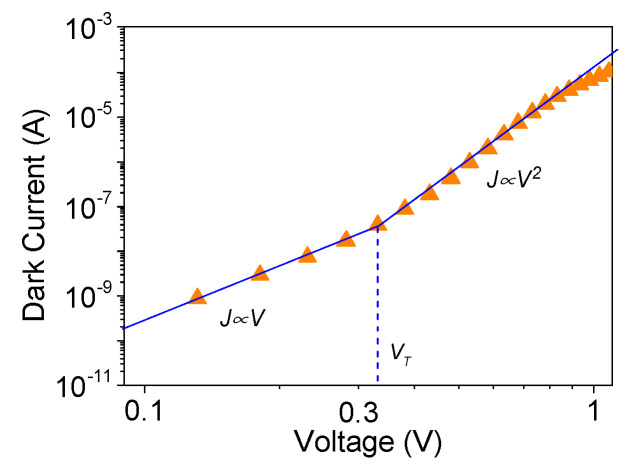
Double logarithmic plot of the dark current vs. voltage characteristic of a typical Si/GaN NW/P3HT:PC_71_BM structure. For comparison, J ∝ V and J ∝ V^2^ to V^3^ dependences are shown by lines. For lower voltages, the photocurrent (orange triangles) shows ohmic behavior, which turns into a V^2^ to V^3^ voltage dependence after threshold voltage V_T_ = 0.33 V.

The double logarithmic plot of dark current for the typical structure is illustrated in Figure 3. A kink-like transition from ohmic to space charge limited behavior is observable in accordance with the Mott–Gurney model, with threshold voltage V_T_ = 0.33 V [29]. Noh et al. have reported similar results while studying the charge transfer mechanism in planar P3HT/GaN structures [13]. The smoothness of the transition points to the presence of traps, which may lead to an increased nonradiative recombination rate.

**Figure 4:**
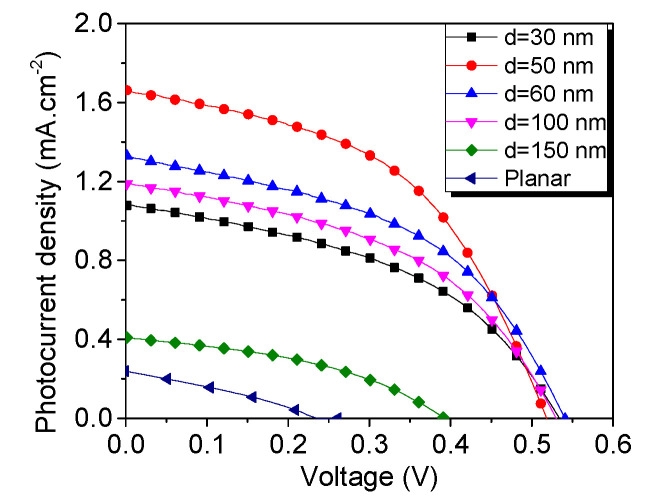
Volt-ampere characteristics of Si/GaN NW/P3HT:PC_71_BM ordered bulk heterostructures with different average nanowire diameters under AM1.5 illumination. Red circles represent the most efficient structure with nanowire diameter d = 50 nm. Color online.

Figure 4 shows the J-V characteristics obtained under AM1.5 spectrum light with irradiance 100 mW·cm^−2^. Nanowire structures are compared with similar ones fabricated on the planar Si/GaN layer. The largest average circuit current density J_sc_ = 1.7 mA.cm^−2^ is achieved on D50 films with V = 0.52 V open-circuit voltage. Compared to the planar structure, this result constitutes an increase by a factor of 4.25. It is immediately obvious that D150 NW arrays show inferior photovoltaic performance, while planar GaN shows the lowest performance, probably due to the lack of boost originating from the nanomorphology [30]. Series resistance R_s_ could be used as a metric pointing to the morphology at which nanowire architecture starts to play a decisive role. While samples with nanowire diameters from 30 to 100 nm all show series resistance between 140 and 250 Ω, the series resistance of D150 and planar structures is significantly higher, at 655 and 695 Ω, respectively. This difference in R_s_ is explained by a combination of two factors: complicated polymer penetration into dense nanowall morphology and almost planar geometry of NW array, which results in planar-like behavior concerning light scattering.

**Figure 5:**
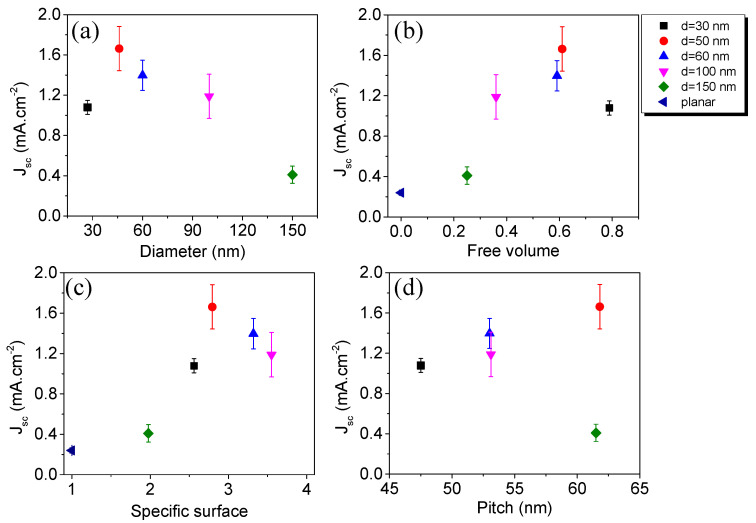
Short circuit current density variation of Si/GaN NW/P3HT:PC_71_BM ordered heterostructures as a function of GaN NW morphological parameters: (**a**) average nanowire diameter; (**b**) free volume of active layer; (**c**) specific surface; (**d**) pitch. Color online.

A systematic study of the effect that the NW morphology has on the Si/GaN NW/P3HT:PC_71_BM photovoltaic performance parameters was carried out under illumination. Results are listed in Table 1. Dependences of J_sc_ on GaN nanostructure morphology are illustrated in Figure 5. J_sc_ is small for average nanowire diameter d = 30 nm, as nanowire conductivity is reduced due to increased surface recombination caused by the lower recombination barrier at the NW surface. The photocurrent shows a maximum for NW diameter d = 50 nm, after which it starts to decline as potential photocurrent enhancement is outweighed by losses due to the decrease in the volume of the organic absorber. A similar decline in J_sc_ for higher values of specific surface illustrated in Figure 5d is explained by the increased recombination rate at the larger NW surface. The optimum free volume and specific surface for self-assembled PAMBE GaN NWs on silicon embedded into P3HT:PC_71_BM are found to be equal to 0.6 and 2.8, respectively. Layers with similar nanowire pitch exhibit different J_sc_ values due to the difference in specific surface.

**Figure 6:**
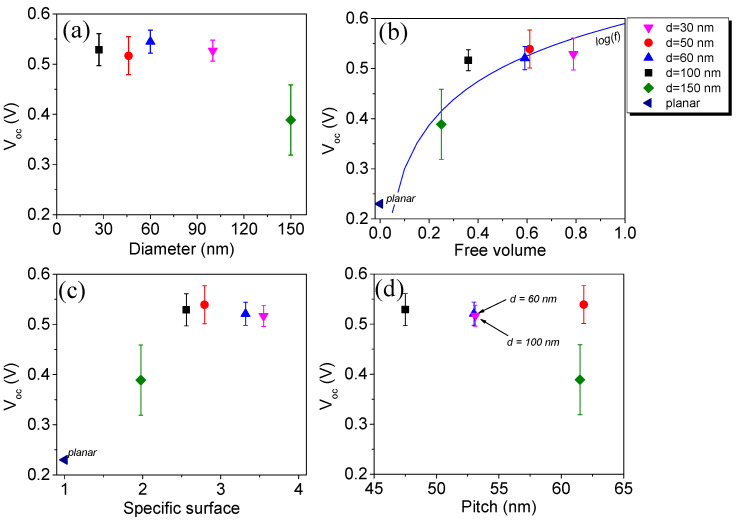
Open circuit voltage variation of Si/GaN NW/P3HT:PC_71_BM ordered heterostructures as a function of GaN NW morphological parameters: (**a**) average diameter; (**b**) free volume—log(f) plot is shown for comparison; (**c**) specific surface; (**d**) pitch. Color online.

Figure 6 shows variations in open-circuit voltage as a function of GaN NW morphology. The V_oc_ vs. free volume rises from V = 0.23 V for the planar sample, for which free volume is 0, to its maximum V = 0.54 volt for the D60 film. The behavior of V_oc_ is consistent with that predicted by the reciprocity relation [31] for nanowire solar cells. In film morphology terms, this relation gives open circuit voltage enhancement caused by transitioning from planar to nanowire architecture as proportional to log(f), where f is the free volume of the nanowire film [32]:

ΔV_oc_ = log(f) k_B_T/e (4)

where k_B_ is Boltzmann constant, T—absolute temperature and e—elementary charge.

This effect is well described for group III-V nanowires embedded in polymer film [33]. The logarithmic guide for the eye is shown, along with measured points, in Figure 6b.

In contrast with J_sc_ behavior, V_oc_ does not decline significantly for NW arrays with the higher specific surface. While surface recombination must still play a significant role, potential V_oc_ loss is most likely outweighed by voltage enhancement, which for NWs with diameter d is proportional to d^3^, while the surface recombination term should be proportional to d^2^.

### 4.3. Mott–Schottky Analysis

**Figure 7:**
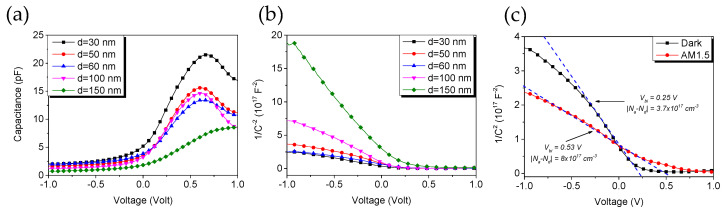
(**a**) Capacitance–voltage characteristics and (**b**) Mott–Schottky 1/C^2^(V) plots of Si/GaN NW/P3HT:PC_71_BM structures. For planar sample V_bi_ = 0.59 V. (**c**) Example of 1/C^2^(V) plot in the dark vs. AM1.5 illumination. Color online.

Capacitance–voltage characteristics and corresponding Mott–Schottky plots are shown in Figure 7a,b. At large reverse bias, capacitance, interpreted as a geometrical capacitance, is the highest for the D30 film and decreases for larger NW diameters. At bias negative to the built-in voltage V < V_bi_, where depletion is modulated by the applied voltage, structures show typical Mott–Schottky behavior, seen as linear segments of 1/C^2^ curves in Figure 7b. Values for built-in voltage V_bi_ and effective doping acquired from fitting are listed in Table 2. V_bi_ values, which depend on acceptor and donor material combination, are generally below those reported in the literature for pristine P3HT:PC_71_BM films [34]. Effective doping |N_a_−N_d_| varies at around 5 × 10^17^ cm^−3^ for samples D30-D60 and decreases after the transition to nanowall morphology. Considering that nanowire pitch in our structures varies between 50 and 60 nm, we conclude that in the dark, the studied samples should be completely depleted, even at low reverse bias. Under AM1.5 illumination (see Figure 7c) the slope of the 1/C^2^ curve changes, indicating a slight increase in carrier concentration. Flatband potential, here equal to V_bi_, is displaced towards positive bias by approximately 0.3 V with respect to the dark conditions. Typically, V_bi_ displacement is explained by a change in surface state occupancy under illumination, although, for pristine P3HT:PCBM films, V_bi_ shifts towards negative voltage. In the literature, this behavior is explained by photogenerated minority carrier accumulation at the contact due to slow kinetics of charge transfer through the surface state [35]. This is, apparently, not the case for Si/GaN NW/P3HT:PCBM films and such heterostructures could be used to overcome the performance limitation caused by carrier injection inhibition typical for organic solar cells.

It has to be mentioned that the validity of standard MS analysis, originally developed for inorganic semiconductors, for bulk heterojunction structures has been called into question since values calculated from MS depend on the measured structure design [36]. Consequently, an expanded model needs to be developed to analyze the behavior of the Si/GaN NW/P3HT:PC_71_BM structure.

## 5. Conclusions

The GaN nanostructures embedded in the P3HT:PC_71_BM blend are investigated as ordered electron acceptors of hybrid organic–inorganic heterostructures fabricated on silicon. In this configuration, P3HT acts as a light absorber and GaN NWs facilitate improved charge transfer to the silicon substrate. The photovoltaic performance of the structure shows dependence on nanowire array morphology. Short circuit current increases by a factor of 4.25 compared to planar Si/GaN/P3HT:PC_71_BM structures, while the open-circuit voltage increases by 0.32 V. Such enhancement is achieved for NW diameters d = 50 nm, for structures with a free volume of 0.6 and specific surface of 2.8. The results of Mott–Schottky analysis of capacitance–voltage dependences are reported. The results show that GaN NWs could be used to integrate P3HT-based organic active layers with novel multilayer Si-based solar cells.

## Figures and Tables

**Table 1:** Photovoltaic performance parameters of Si/GaN NW/P3HT:PC_71_BM heterostructures for nanowire diameters 30, 50, 60, 100, 150 nm.

Structure	J_sc_ (mA·cm^−2^)	V_oc_ (V)	Fill Factor	PCE (%)	R_s_ (Ω)	R_sh_ (Ω)
D30	1.09 (±0.07)	0.53 (±0.03)	0.44	0.25	200	1460
D50	1.69 (±0.22)	0.52 (±0.04)	0.49	0.42	140	1200
D60	1.33 (±0.15)	0.54 (±0.02)	0.44	0.34	250	1340
D100	1.18 (±0.22)	0.52 (±0.02)	0.49	0.42	155	1520
D150	0.41 (±0.09)	0.39 (±0.07)	0.41	0.07	695	1280
planar	0.24 (±0.02)	0.23 (±0.01)	0.27	2 × 10^−4^	655	1280

**Table 2:** Mott–Schottky analysis results of Si/GaN NW/P3HT:PC_71_BM heterostructures for nanowire diameters 30, 50, 60, 100, 150 nm.

Structure	V_bi_ (V)	|N_a_−N_d_|(cm^−3^)
D30	0.24	6.1 × 10^17^
D50	0.25	5.9 × 10^17^
D60	0.36	5.7 × 10^17^
D100	0.3	2.6 × 10^17^
D150	0.32	7.1 × 10^16^
